# Electrochemical Studies of Stainless Steel and Stainless Steel-TiO_2_ Composite in Reference to Molten Aluminum Alloy Using a Solid-State BaCO_3_ Electrolyte

**DOI:** 10.3390/ma15196723

**Published:** 2022-09-27

**Authors:** Piotr Malczyk, Marcel Mandel, Tilo Zienert, Christian Weigelt, Lutz Krüger, Jana Hubalkova, Gert Schmidt, Christos G. Aneziris

**Affiliations:** 1 Institute of Ceramics, Refractories and Composite Materials, TU Bergakademie Freiberg, 09599 Freiberg, Germany; 2 Institute of Materials Engineering, TU Bergakademie Freiberg, 09599 Freiberg, Germany

**Keywords:** corrosion, high-temperature electrochemical method, metal matrix composite, liquid aluminum alloy, solid-state electrolyte, potentiodynamic polarization, differential electrode potential

## Abstract

The influence of TiO_2_ addition on the high-temperature electrochemical characteristics of stainless-steel-based materials was investigated by means of differential potential measurement, electrochemical polarization and impedance spectroscopy. A new three-electrode approach was utilized which incorporated a liquid aluminum alloy AlSi7Mg0.3 as the reference electrode, barium carbonate BaCO_3_ as the solid-state electrolyte, and stainless steel or a stainless steel-TiO_2_ composite as the working electrode. The potential differences between the steel-based working electrodes and the liquid-aluminum-alloy reference electrode were measured for 85 h throughout the whole experiment, including the heating and cooling period. The experiments were performed at 850 °C. The determination of the high-temperature open circuit potential (*E*_Corr_) in reference to the liquid aluminum alloy was carried out via potentiodynamic polarization. The polarization-related changes in the impedance characteristics were evaluated by the correlation of impedance responses before and after the polarization. The addition of 40 vol% TiO_2_ resulted in a reduction in the potential of the steel-TiO_2_ composite and led to the formation of a more uniform electrode–electrolyte interface. The reaction products on the surface of the working electrodes were investigated by means of SEM/EDS and XRD. They consisted of mixed oxides within the Fe-O, Ba-Fe-O and Ba-Cr-O systems.

## 1. Introduction

Liquid aluminum is one of the most reactive metals; it can dissolve or corrode other metal-based materials and react with numerous ceramics. For contact with liquid aluminum alloys, functional components based on refractory ceramics are applied. The conventional manufacturing technology for refractory ceramics with drying and prefiring steps is very time-consuming and can lead to the initiation of internal stresses resulting in failure of the refractory components. Dense refractory ceramics are characterized by poor thermal shock resistance, which entails reduced lifecycles. Moreover, conventional technologies of refractory ceramics do not enable the manufacture of complex-shaped products. Therefore, alternative materials with superior thermo-mechanical and corrosion properties need to be developed.

Ceramic-reinforced steel-based metal matrix composites exhibit the potential to fulfill the necessary requirements in this application area. Although liquid aluminum is able to dissolve steel, recent studies have revealed that the addition of 40 vol% of selected ceramic particles to steel can significantly increase the corrosion resistance of steel–ceramic composites against aluminum and aluminum alloys [1]. Good thermal shock and corrosion resistance linked to high ductility and good machinability predestinate steel–ceramic composites for application in the aluminum industry. Moreover, good machinability and higher ductility make steel–ceramic composites suitable materials for tailor-made substitutes of refractory products.

The corrosive action of aluminum is very complex and requires a comprehensive, interdisciplinary approach, taking not only its chemical reactivity and wettability but also electrochemical phenomena occurring at the contact interface with steel–ceramic composite materials into consideration. For this purpose, the determination of the differential potential between the liquid aluminum alloy and the steel–ceramic composite at the aluminum processing temperature of 850 °C is essential [1]. The difference in the electrochemical potential between two electrodes determines the reaction driving force and thus the corrosion rate. The corrosion rate (*CR*) is proportional to the mass loss rate (*MR*) and thus to the corrosion current *I*_Corr_, according to Equation (1) [2,3,4]:

(1) *CR* ∝ *MR* = K_2_ × 10^−3^(*I*_Corr_) × *EW*

where the *MR* is the mass loss per day (g·m^−2^), *EW* is the equivalent of the corroding material (g·mol^−1^) and K_2_ is the material constant for daily mass loss rate (mg·m^2^·A^−1^·dm^−2^).

The corrosion current is proportional to the potential difference Δ*E* between two electrodes, following Ohm’s law (2) [3,4]:

(2) $$I_{\mathrm{Corr}}=\frac{\Delta E}{R_{T}}$$

where *R*_T_ is equal to the total electrical resistance of an electrochemical cell.

For the assessment of the reaction driving force under close-to-standardized conditions, the calculation of the standard electrode potentials (*E*^0^) is commonly performed using the following equation:

(3) $$\Delta E=E_{M1}^{0}-E_{M2}^{0}$$

where $E_{M1}^{0}$ and $E_{M2}^{0}$ are the standard electrode potentials of electrode materials M1 and M2, respectively [2,5,6,7].

At elevated temperatures, the potential difference is determined by the measurement of the cell potential (*E*_Cell_):

(4) $$E_{\mathrm{Cell}}=\Delta E=E_{M1}^{T}\;–\;E_{M2}^{T}$$

or by considering the voltage drop at the electrolyte (*U*_e_):

(5) $$E_{\mathrm{Cell}}=\Delta E-U_{e}=E_{M1}^{T}\;–\;E_{M2}^{T}-I_{\mathrm{Corr}}R_{e}$$

where $E_{M1}^{T}$ and $E_{M2}^{T}$ are the potentials of the M1 and M2 electrodes at the temperature T, *R_e_* is the electrical resistance of the electrolyte and *I*_Corr_ is the open circuit current flowing through the electrochemical cell [8].

The determination of the cell potential is often carried out by simple voltage measurements [2,5,8,9,10]. For the three-electrode cell, the cell potential—the potential difference between the working and reference electrodes—is indicated by the open circuit potential (*E*_Corr_), determined by potentiodynamic polarization [2,10,11]. For this purpose, under standardized conditions, with stringent criteria regarding temperature, atmospheric pressure, the material of the reference electrode, electrolyte solution concentration, etc., a simple difference between the standard electrode potentials of both materials is commonly used [6,7,8,10,11]. However, the determination of the cell potential and the investigation of the electrochemical behavior of electrodes under highly demanding thermal and physical conditions are very complicated and require the development of novel, suitable experimental methods.

Electrochemical studies at elevated temperatures are usually performed at temperatures from 60 to 400 °C [12,13,14,15,16,17,18]. Such temperatures are far below the temperature of interest, in particular that of 850 °C—the common temperature of heat-retaining aluminum furnaces [1]. The corrosion of steel-based materials at temperatures exceeding the melting point of aluminum alloys has been rarely investigated. Sah et al. [19] studied the corrosion behavior of austenitic steels in molten alkali carbonates M_2_CO_3_ (M = K, Li, Na). Subari et al. [20] investigated the corrosion of steels in contact with molten salt eutectics of KNO_3_, LiNO_3_, NaCl and NaNO_3_. Nevertheless, for electrochemical experiments with aluminum alloys at 850 °C, the application of molten salts is excluded due to the fact that the electrolyte and the aluminum alloy will both remain liquid, resulting in uncontrollable two-way diffusion. Moreover, the application of molten salts at the electrolyte is hampered by their susceptibility to decomposition below their melting point, requiring very strict testing conditions (e.g., maintenance of the vapor pressure of the salt during the process) [21,22].

The determination of the high-temperature differential potential for the solid–liquid electrode pair requires the application of a solid-state electrolyte to prevent undesired infiltration of the liquid electrode within the electrochemical cell. The applied electrolyte should be characterized by sufficient thermal stability, solid-state ion conductivity and a melting point above the testing temperature. Moreover, the electrolyte should be inert to the construction material of the electrochemical cell. For electrochemical experiments with liquid aluminum alloys as reference electrodes, barium carbonate BaCO_3_ can be successfully applied as the solid-state electrolyte. It decomposes at 1360 °C, without an ordinary melting point. At a temperature of 811 °C, BaCO_3_ undergoes a polymorphic transformation from the orthorhombic (Pmcn) to the trigonal (R3m) crystal structure, with an enhancement of Ba^2+^ cation mobility and thus ion conductivity [23,24]. Moreover, BaCO_3_ does not react with alumina-based refractory ceramics at a temperature of 850 °C, i.e., alumina can be used as a construction material for the electrochemical cell.

To the best of the authors’ knowledge, there is no information about the corrosion of steel-based materials investigated using solid electrolyte and liquid reference electrodes. Furthermore, no electrochemical studies seeking to determine the differential electrode potentials of dissimilar metallic electrodes at elevated temperatures were found in the literature. This study addresses the effect of TiO_2_ addition on the high-temperature electrochemical behavior and the differential potential of stainless steel 316L in reference to the liquid aluminum alloy AlSi7Mg0.3 at 850 °C using a solid-state BaCO_3_ electrolyte.

## 2. Experimental

### 2.1. Materials and Manufacturing

Within the scope of this study, two electrodes—pure steel and steel-TiO_2_—were manufactured and tested. The plain stainless-steel electrode was manufactured from gas-atomized stainless-steel powder 316L-FeCr18Ni10Mo3 (TLS Technik, Bitterfeld-Wolfen, Germany). The powder mixture for manufacturing the steel-TiO_2_ composite electrode consisted of 60 vol% stainless-steel powder and 40 vol% TR HP-2 rutile TiO_2_ (Sachtleben Chemie, Duisburg, Germany). Hereinafter, the electrode samples are named “316L” and “316L40TiO_2_”, referring to the plain steel and the composite electrodes, respectively. The as-delivered composition of the steel powder is described in Table 1. Table 2 contains the percentiles of particle size distributions as well as the true densities of the raw materials.

In order to obtain a homogeneous distribution of TiO_2_ particles within the 316L40TiO_2_ steel–ceramic composite, the powder mixture was dry mixed for 120 min by means of a roller mill using 3 mm and 5 mm stainless-steel milling balls. The mass of added milling balls was related to the mass of the powder mixture and amounted to 21% for 5 mm balls and 16% for 3 mm balls. After milling, a liquid temporary binder (2.5 wt% related to the mass of the powder mixture) was added to prepare a pressing mass. The bonding additive consisted of the 25% polyvinyl alcohol-based binder OPTAPIX PAF 35 (Zschimmer & Schwarz, Lahnstein, Germany) and 75% deionized water. The plain stainless steel did not require previous homogenization and was therefore prepared without a dry mixing process.

Prepared masses were pressed to prisms with dimensions of 7 mm × 7 mm × 70 mm using a uniaxial press (Rucks, Glauchau, Germany). The pressing procedure had a consolidation pressure of 100 MPa preceded by two air-degassing steps (30 and 60 MPa for 1 s). After uniaxial pressing, all samples were dried at 110 °C for 24 h in a convection drying oven.

**Figure 1:**
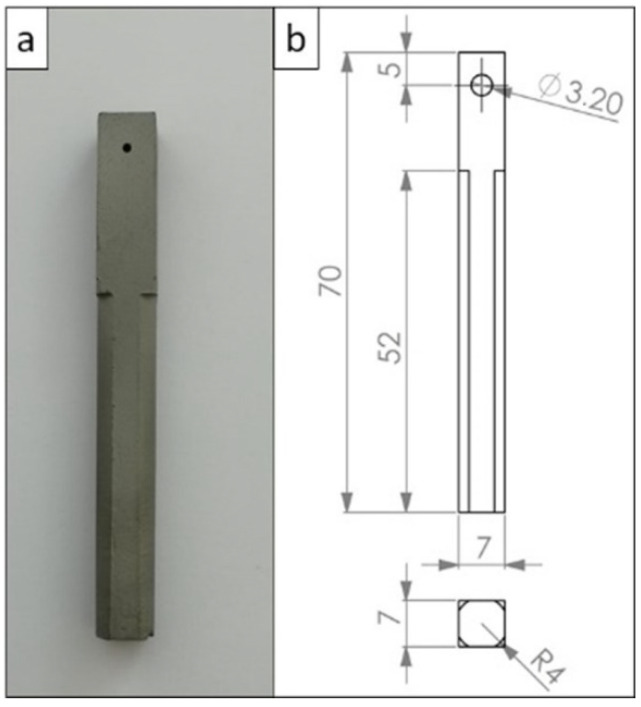
Working electrode for the electrochemical investigations: (**a**) a photograph of the prepared sample; (**b**) a technical drawing with geometrical dimensions of the sample in mm.

The binder removal was carried out in a debinding furnace (Xerion, Berlin, Germany) with a heating rate of 2 K·min^−1^ to 200 °C, followed by a heating rate of 0.5 K·min^−1^ from 200 to 450 °C and a holding time of 30 min at 450 °C. The samples were then cooled at a cooling rate of 0.5 K·min^−1^. After debinding, the samples were sintered at 1350 °C for 2 h using a graphite-lined furnace (Xerion, Berlin, Germany) under argon atmosphere with constant heating and a cooling rate of 5 K·min^−1^. The sintered samples were subjected to the final preparation by the drilling of wire mounting holes, machining to the desired shapes and removal of sinter oxide skins from the surfaces. Figure 1 presents a photograph and technical drawing of the electrode used for the electrochemical testing.

The electrochemical experiments were carried out using common silicon pre-eutectic AlSi7Mg0.3 casting aluminum alloy (TRIMET Aluminum, Essen, Germany) as the liquid reference electrode. The as-delivered composition of the aluminum alloy is described in Table 3.

The selection of BaCO_3_ as the solid electrolyte was based on preliminary differential scanning calorimetry and thermogravimetry measurements (DSC/TG) taken while investigating the applicable temperature range of the salt in consideration of the designated temperature of the electrochemical experiment (850 °C). The measurement was carried out with synthetic air flushing (19.9–21.9% O_2_, 2 ppm H_2_O, Praxair Industriegase, Germany) up to 1000 °C, with a heating and cooling rate of 10 K·min^−1^, using a STA 409 PC calorimeter (Netzsch, Selb, Germany).

### 2.2. Electrochemical Experiments

**Figure 2:**
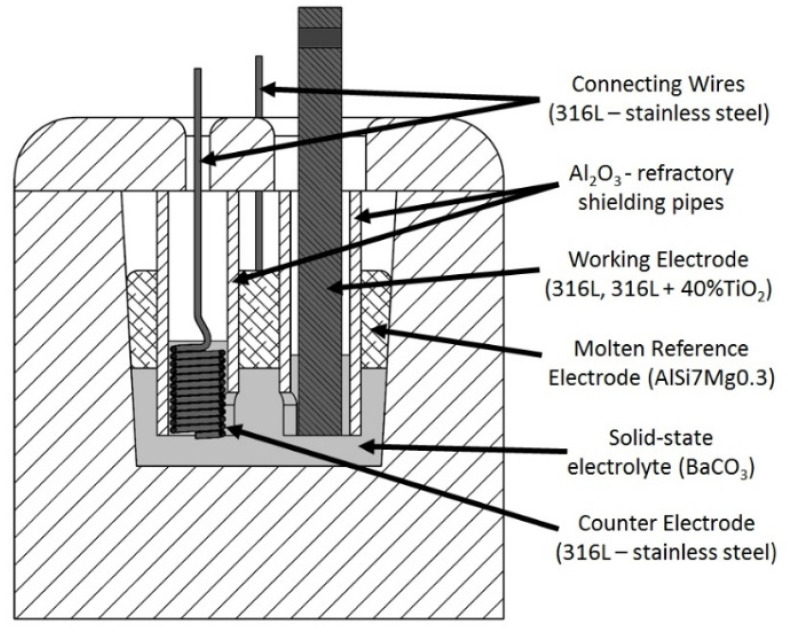
Schematic drawing of the high-temperature three-electrode cell with the 316L/molten AlSi7Mg0.3 reference electrode and the 316L stainless-steel counter electrode.

The crucible and the lid of the three-electrode electrochemical cell were manufactured from alumina-based refractory ceramics with silica-free binder, according to Dudczig [25], and sintered at 1600 °C for 4 h under air atmosphere with heating and cooling rates of 3 K·min^−1^. The lid was equipped with three holes—two for connecting wires and one for the working electrode. A schematic drawing of the three-electrode electrochemical cell is presented in Figure 2.

For each experiment, 40 g of BaCO_3_ powder (99% BaCO_3_, Carl Roth, Karlsruhe, Germany) was put into the crucible and compacted with the aluminum alloy reference electrode (RE). The RE had a cylindrical form, 40 mm in diameter and 15 mm in height, and had two predrilled holes with diameters of 12 mm for the introduction of the working and counter electrodes. Both the working electrode (WE) and the counter electrode (CE) were inserted into the BaCO_3_ through the holes using alumina refractory shielding pipes to prevent direct contact with the aluminum alloy (RE). For both systems, the finger-shaped WE had a diameter of 8 mm and a surface of 3.4 cm^2^ exposed to the BaCO_3_. The spring-shaped CE was made of 1 mm-diameter steel wire, increasing the exposed surface of the electrode and inhibiting the CE saturation. To avoid any influence of dissimilar interfaces on the measurement of the differential potential, the CE as well as the wires were made of 316L stainless steel, matching the matrix material of the WE. The prepared cell was installed in an alumina retort and placed into the furnace.

The electrochemical experiment was carried out at 850 °C for 85 h, with a heating and cooling rate of 1 K·min^−1^. During the whole experiment, the temperature inside the retort and the potential differences between WE and RE, as well as between WE and CE, were measured using the ALMEMO 2290-8 data logger (Ahlborn, Germany). The recording of both potential differences during the heating and holding provided information about the transition of the system into the steady state. After reaching the steady state, i.e., 30 h at 850 °C, the electrical potential differences were determined and the potentiodynamic polarization and impedance spectroscopy measurements were performed. The polarization and impedance behavior were analyzed using a Reference 600 potentiostat (Gamry Instruments, Warminster, PA, USA). To analyze the influence of the polarization process on the impedance response of the system, the impedance behavior was investigated before and after the polarization test. The potentiodynamic polarization was carried out with a scan rate of 0.5 mV·s^−1^ in the range of 0.2 V ≤ AlSi7Mg0.3/316L ≤ 2.6 V. The impedance measurements were performed in a frequency range from 50·10^−2^ Hz to 5·10^5^ Hz, using a sinusoidal potential wave with an amplitude of 50 mV.

**Figure 3:**
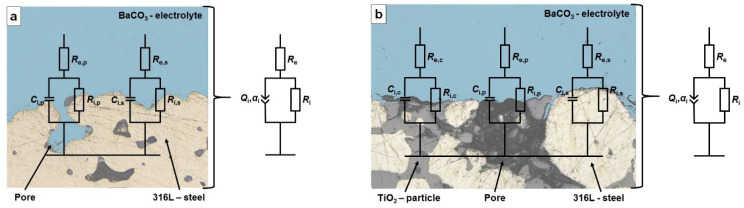
Equivalent circuits along with schematic illustrations based on LSM micrographs of the considered surface components (steel particles, pores and ceramic particles) of both electrochemical systems: (**a**) 316L (WE); (**b**) 316L40TiO_2_ (WE). *R*_e_, *R*_i_, *Q*_i_ and *α*_i_ represent local and global parameters for the electrolyte and electrolyte–electrode interfaces.

For the characterization of impedance response and distribution of the charge, an equivalent model with surface distribution along the electrolyte–electrode interface was selected [26,27,28,29,30]. To comprehensively describe the impedance response of the system, the constant phase element (CPE) as an equivalent for the distributed capacity was utilized [26,27,29]. The impedance of the constant phase element (*Z*_CPE_) is expressed by Equation (6):

(6) $$Z_{\mathrm{CPE}}=\frac{1}{Q{\left(j\omega \right)}^{\alpha }}$$

where *Q* is the CPE parameter and *α* is the CPE exponent, which describe the capacitive characteristics of the electrode, and jω is the imaginary unit multiplied by angular frequency. Exemplary models of the surface distributions of both the 316L and 316L40TiO_2_ electrode surfaces are presented in Figure 3.

The effective capacitance (*C*_eff_) and corresponding time constant (*τ*_eff_) of the circuit equivalent with surface distribution arise according to Equations (7) and (8) [26,27,28,31]:

(7) $$C_{\mathrm{eff}}={\left[Q{\left(\frac{1}{R_{e}}+\frac{1}{R_{i}}\right)}^{\alpha -1}\right]}^{\frac{1}{\alpha }}$$

(8) $$\tau_{\mathrm{eff}}=\frac{R_{e}R_{i}}{R_{e}+R_{i}}C_{\mathrm{eff}}$$

where *R*_e_ is the electrolyte resistance and *R*_i_ is the global resistance of the electrode–electrolyte interface.

### 2.3. Analysis of the Electrode–Electrolyte Interfaces

After completion of the electrochemical experiment, the 316L and 316L40TiO_2_ working electrodes were carefully removed from the electrochemical cell and their surfaces were analyzed.

The investigation of the surface of both the 316L and 316L40TiO_2_ working electrodes after the experiment was performed with a VHX-3000 digital optical microscope and a VK/X1000 laser scanning microscope (both manufactured by Keyence, Neu-Isenburg, Germany). The analysis of the reaction products on the surfaces of the electrodes were carried out using an XL 30 ESEM FEG scanning electron microscope (FEI/Philips, Eindhoven, The Netherlands).

The phase analysis of the electrode–electrolyte reaction products was carried out using the X-Ray Diffractometer Empyrean DY1946 (Malvern Panalytical, Kassel, Germany) with 40 kV and 40 mA power and Cu Kα radiation and evaluated with Rietveld analysis. Grazing incidence X-ray diffraction (GIXD) of the flat sample surface with an incidence angle of Ω = 3° was used to investigate the reaction products at the surfaces of the working electrodes. Moreover, to study the possible decomposition of BaCO_3_ in contact with the liquid aluminum alloy, phase analysis of the electrolyte after the experiment was performed by powder diffraction using Bragg–Brentano optics.

## 3. Results and Discussion

### 3.1. Thermal Analysis of BaCO_3_

**Figure 4:**
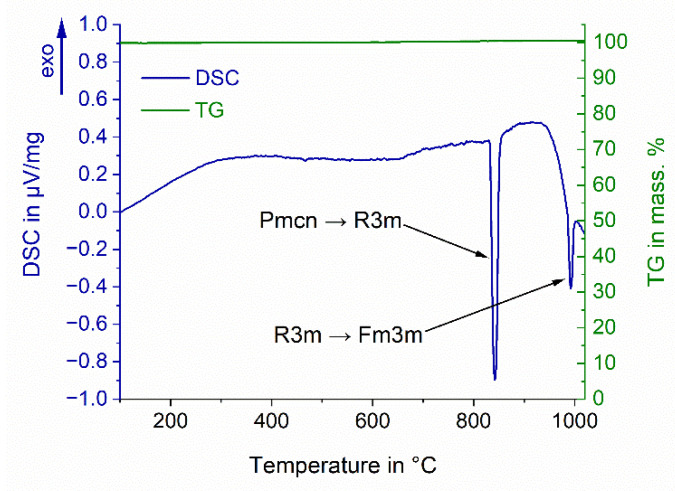
The DSC/TG measurement of BaCO_3_ up to 1000 °C (heating rate of 10 K·min^−1^).

The results of the DSC/TG analysis of BaCO_3_ to estimate the applicable temperature range of the electrolyte are presented in Figure 4.

The DSC analysis revealed two endothermic peaks during the heating of BaCO_3_ up to 1000 °C. The first endothermic peak indicates the polymorphic transformation of the salt from the orthorhombic (Pmcn) to the trigonal (R3m) crystal structure, with an onset point at 820 °C [23]. The second endothermic peak indicates continuous transformation of BaCO_3_ from the trigonal (R3m) to the cubic (Fm3m) structure, with an onset point at 940 °C. No decomposition of the salt during the polymorphic transformations was observed. Due to the increased mobility of Ba^2+^ cations in the trigonal (R3m) crystal structure of BaCO_3_, the salt can be successfully applied as the solid electrolyte in the temperature range between 830 and 930 °C [23].

### 3.2. Differential Electrode Potential

**Figure 5:**
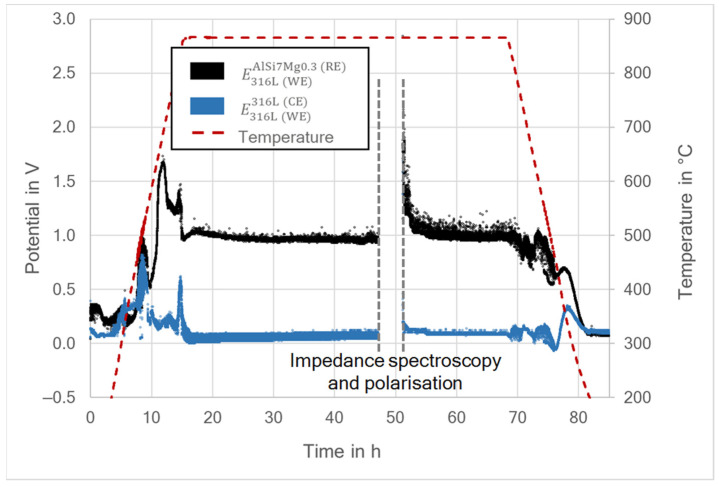
Recorded differential potentials of the 316L (WE)–AlSi7Mg0.3 (RE)–316L (CE) system.

**Figure 6:**
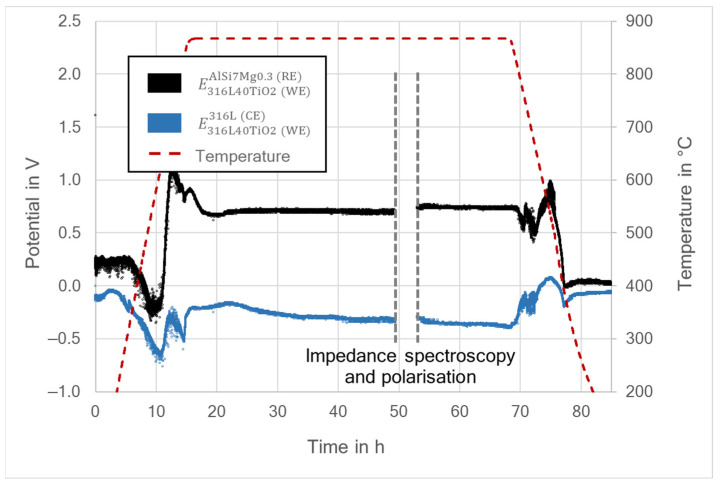
Recorded differential potentials of the 316L40TiO_2_ (WE)–AlSi7Mg0.3 (RE)–316L (CE) system.

The potential differences between WE and RE, as well as between WE and CE, were measured throughout the whole experiment, including the heating and cooling process. The differential potential is expressed by $E_{M1}^{M2}$, with M1 being the working electrode and M2 the material of the electrode to which the potential is measured [2,5]. Therefore, $E_{316L\left(\mathrm{WE}\right)}^{\mathrm{AlSi}7\mathrm{Mg}0.3\left(\mathrm{RE}\right)}$ refers to the differential potential of the 316L (WE) measured against the aluminum alloy AlSi7Mg0.3 (RE), while $E_{316L\left(\mathrm{WE}\right)}^{316L\left(\mathrm{CE}\right)}$ is the differential potential of the 316L (WE) measured against the stainless-steel 316L (CE). Similarly, for the 316L40TiO_2_ (WE) the differential potentials measured against the RE and CE were designated as $E_{316L40\mathrm{TiO}2\left(\mathrm{WE}\right)}^{\mathrm{AlSi}7\mathrm{Mg}0.3\left(\mathrm{RE}\right)}$ and $E_{316L40\mathrm{TiO}2\left(\mathrm{WE}\right)}^{316L\left(\mathrm{CE}\right)}$, respectively. Figure 5 and Figure 6 present the results of the differential potential measurements for both systems. The grey, vertical, dashed lines indicate the time span during which the polarization measurements along with the impedance spectroscopy were carried out.

During the heating and cooling, the differential potential measurements revealed multiple peaks related to the stabilization of the system, the polymorphic transformation of the BaCO_3_ and also the melting and solidification of the aluminum alloy (RE). After approximately 30 h at 850 °C, both systems reached a steady state and the differential potentials could be determined. The measured differential potentials for 316L (WE) yielded values ranging from 0.83 to 1.03 V for $E_{316L\left(\mathrm{WE}\right)}^{\mathrm{AlSi}7\mathrm{Mg}0.3\left(\mathrm{RE}\right)}$ and from 0.04 to 0.11 V for $E_{316L\left(\mathrm{WE}\right)}^{316L\left(\mathrm{CE}\right)}$ (Figure 5). In comparison, the values for 316L40TiO_2_ (WE) ranged from 0.69 to 0.71 V for $E_{316L40\mathrm{TiO}2\left(\mathrm{WE}\right)}^{\mathrm{AlSi}7\mathrm{Mg}0.3\left(\mathrm{RE}\right)}$ and from −0.35 to −0.31 V for $E_{316L40\mathrm{TiO}2\left(\mathrm{WE}\right)}^{316L\left(\mathrm{CE}\right)}$ (Figure 6). Hence, there was a significant difference of 0.23 V on average between $E_{316L\left(\mathrm{WE}\right)}^{\mathrm{AlSi}7\mathrm{Mg}0.3\left(\mathrm{RE}\right)}$ and $E_{316L40\mathrm{TiO}2\left(\mathrm{WE}\right)}^{\mathrm{AlSi}7\mathrm{Mg}0.3\left(\mathrm{RE}\right)}$. Furthermore, the smaller amplitude scatter indicated better stability of the system with 316L40TiO_2_ (WE). This may be due to the more uniform WE–electrolyte reaction interface for the 316L40TiO_2_ sample. The recorded potential differences after the impedance spectroscopy and polarization experiments revealed that the system based on the 316L stainless-steel working electrode was brought out of the steady state by the performed measurements (Figure 5). The stabilization of $E_{316L\left(\mathrm{WE}\right)}^{\mathrm{AlSi}7\mathrm{Mg}0.3\left(\mathrm{RE}\right)}$ lasted over 4 h, decreasing from 2 V into the range of 0.9–1.2 V; hence, its amplitude scatter increased. In comparison, $E_{316L40\mathrm{TiO}2\left(\mathrm{WE}\right)}^{\mathrm{AlSi}7\mathrm{Mg}0.3\left(\mathrm{RE}\right)}$ (Figure 6) exhibited no considerable changes and stabilized at a level between 0.73 and 0.76 V after the polarization and impedance spectroscopy measurements.

### 3.3. Impedance Spectroscopy and Potentiodynamic Polarization

**Figure 7:**
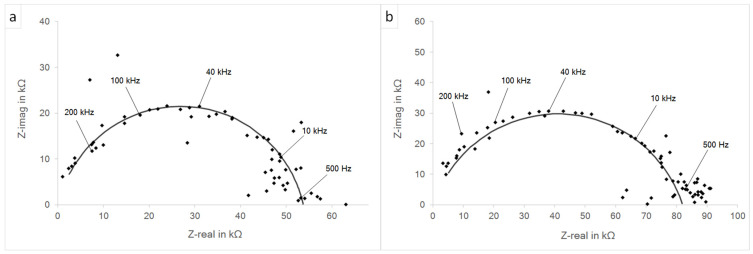
Nyquist plot of the impedance spectroscopy prior to the potentiodynamic polarization: (**a**) 316L (WE); (**b**) 316L40TiO_2_ (WE).

Figure 7 presents the impedance spectra of both investigated systems prior to the potentiodynamic polarization measurements. The derived parameters and calculated values for *C*_eff_ and *τ*_eff_ are summarized in Table 4.

In both material systems, the estimated electrical resistance of the BaCO_3_ solid electrolyte Re was 100 Ω, this being significantly higher compared to commonly used liquid-electrolyte-based solutions [30]. The polarization resistances at the electrode–electrolyte interfaces were 53.6 kΩ and 81.9 kΩ for the systems with 316L (WE) and 316L40TiO_2_ (WE), respectively. The higher polarization resistance of the system with 316L40TiO_2_ (WE) was induced by the presence of TiO_2_ particles at the electrode–electrolyte interface and correlated with the lower reactivity of the composite. The presence of TiO_2_ particles decreased the effective capacitance and thus the effective time constant of the system. The *C*_eff_ and *τ*_eff_ of the system with 316L40TiO_2_ (WE) were 8.74 pF and 0.87 ns, respectively. The *C*_eff_ and *τ*_eff_ of the system with 316L (WE) were 21.56 pF and 2.15 ns, respectively. The *α*_i_ parameters revealed no significant difference and were at the near-capacitive characteristics of the CPE. Both measurements exhibited slight scatter of the collected measurement points, possibly due to the application of BaCO_3_ as the solid-state electrolyte, forcing solid–solid ion transfer and thus affecting the impedance responses of both systems.

**Figure 8:**
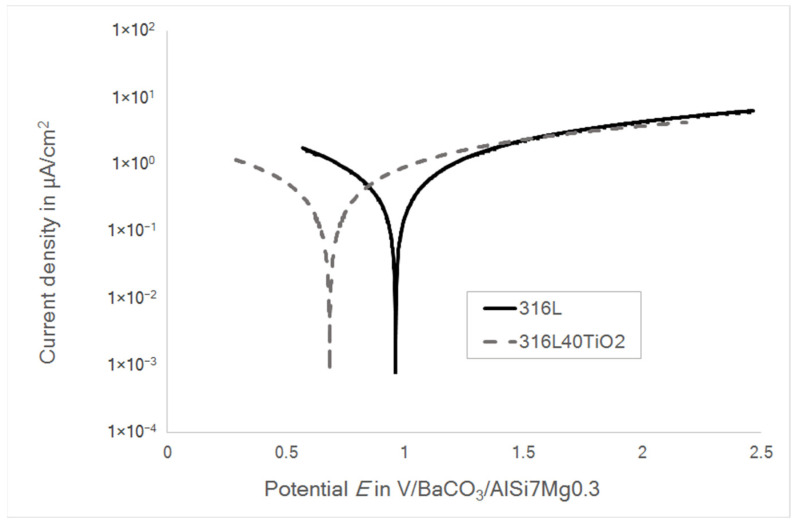
Potentiodynamic polarization of the systems with 316L and 316L40TiO_2_ in reference to the aluminum alloy AlSi7Mg0.3 in BaCO_3_ at 850 °C.

Figure 8 presents the potentiodynamic polarization curves for both the 316L (WE) and 316L40TiO_2_ (WE) systems polarized in reference to the liquid AlSi7Mg0.3 (RE).

The open circuit potential (*E*_Corr_) of the system with 316L (WE) was 0.93 V/BaCO_3_/AlSi7Mg0.3, while that of the system with 316L40TiO_2_ (WE) was 0.71 V/BaCO_3_/AlSi7Mg0.3. These results were in agreement with the results for the differential potentials ($E_{316L\left(\mathrm{WE}\right)}^{\mathrm{AlSi}7\mathrm{Mg}0.3\left(\mathrm{RE}\right)}$ and $E_{316L40\mathrm{TiO}2\left(\mathrm{WE}\right)}^{\mathrm{AlSi}7\mathrm{Mg}0.3\left(\mathrm{RE}\right)}$; cf. Figure 5 and Figure 6), meeting the theory-based expectations [2,5,8,9,10,27]. A slightly lower current density in the system with 316L40TiO_2_ (WE) was induced by the presence of TiO_2_ at the electrode–electrolyte interface, leading to the higher polarization resistance of the electrode (cf. Table 4). It can be stated that the electrical charge in the 316L40TiO_2_ (WE) system was transferred mainly through the conduction of steel particles in the composite.

**Figure 9:**
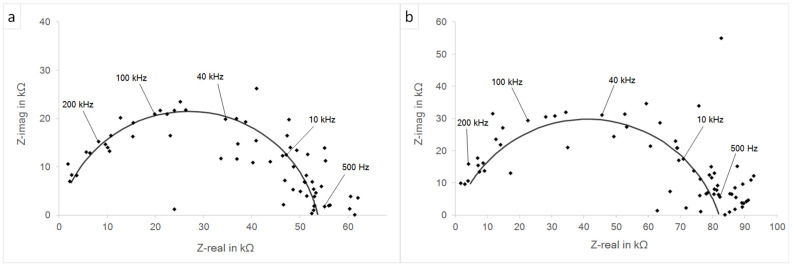
Nyquist plot of the impedance spectra posterior to potentiodynamic polarization: (**a**) 316L (WE); (**b**) 316L40TiO_2_ (WE).

Figure 9 presents the impedance spectra for both investigated systems posterior to the potentiodynamic polarization measurements. The derived parameters and calculated values for *C*_eff_ and *τ*_eff_ are summarized in Table 5.

A comparison of the dispersion of the measurement points from the applied fitting curve prior and posterior to the polarization revealed that both systems were brought out of the steady state. Nevertheless, the curve-fitting parameters were preserved unchanged and thus the impedance fitting results were the same. The *R*_e_ for both systems was 100 Ω. The polarization resistances were 53.6 kΩ and 81.9 kΩ for the systems based on 316L (WE) and 316L40TiO_2_ (WE), respectively. The CPE parameters *Q*_i_ and *α*_i_ as well as the calculated *C*_eff_ and *τ*_eff_ remained unchanged.

Regarding the fact that the open circuit current I_Corr_ and the polarization resistance of the electrolyte were relatively low, the voltage drop caused by the electrolyte was negligible and could be omitted for the estimation of the differential potential. The determined value for the differential potential between the liquid AlSi7Mg0.3 (RE) and 316L (WE) was 0.93 V, while that between the liquid AlSi7Mg0.3 (RE) and 316L40TiO_2_ (WE) was 0.71 V, these results being in agreement with the differential potential measurements presented in Figure 5 and Figure 6.

### 3.4. Analysis of the Electrode Surfaces

**Figure 10:**
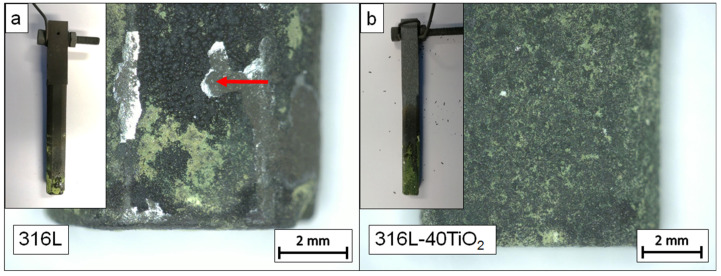
Macrographs and micrographs of working electrodes after the electrochemical experiments in BaCO_3_ at 850 °C: (**a**) 316L (WE); (**b**) 316L40TiO_2_ (WE).

Figure 10 shows photographs and optical micrographs of the surface of working electrodes after the electrochemical experiments.

The surfaces of both working electrodes reacted with the electrolyte during the electrochemical experiments. On both working electrodes, similar green- and brown-colored reaction products were observed. The surface of the 316L40TiO_2_ (WE) was characterized by a more uniform distribution of the reaction products (Figure 10b), while their distribution on the 316L (WE) was less uniform, with distinctive green- and brown-colored clusters (Figure 10a). Moreover, for the 316L (WE), detachment of the reaction layer was observed (Figure 10a, red arrow). The thickness of the detached reaction layer determined by the VK/X1000 Laser Scanning Microscope (Keyence, Neu Isenburg, Germany) using MultiFileAnalyser was approximately 64 µm. No detachment of the reaction layer was observed for the 316L40TiO_2_ (WE).

**Figure 11:**
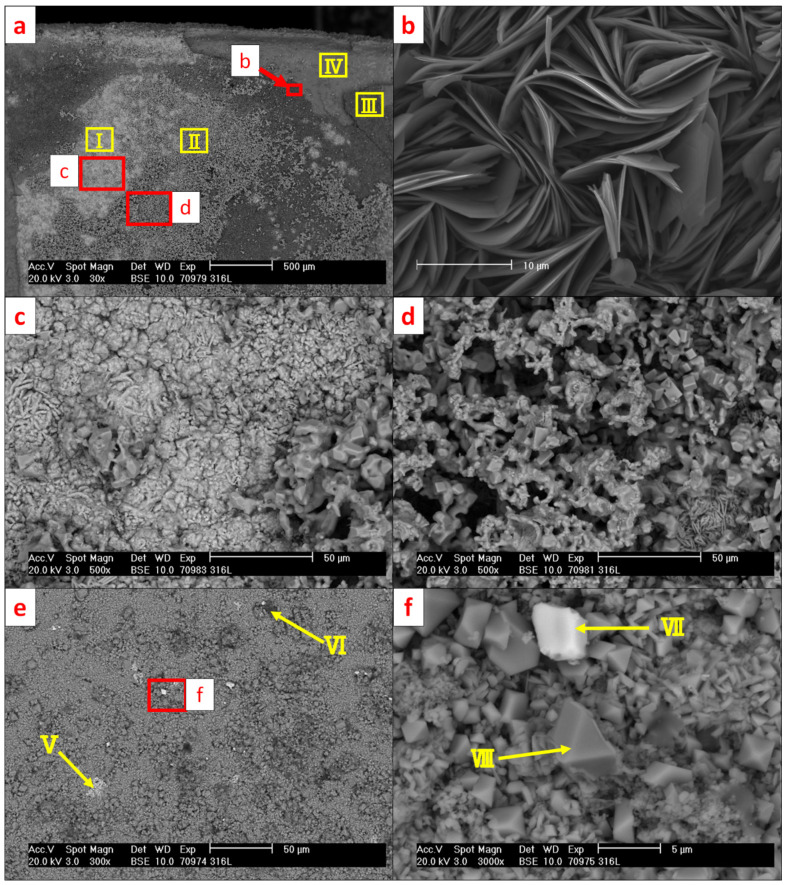
SEM micrographs (BSE mode) of 316L (WE) after the electrochemical experiment at 850 °C in the BaCO_3_ electrolyte with molten AlSi7Mg0.3 (RE): (**a**) overview; (**b**) brown-colored leaf-shaped Fe-O nanoparticles; (**c**) green-colored BaCrO_4_; (**d**) green-colored BaFe_12_O_19_ with Cr-Mn-Fe mixed oxides, (**e**) agglomerates and crystal phases with indicated Fe-Cr-Ni-Si and Al_2_O_3_, (**f**) Cr-Mn-Fe and Ba-Fe-Cr mixed oxide crystals. Red frames indicate the regions of the magnified views of the micrographs; yellow frames and arrows indicate the areas of the EDS scans.

**Figure 12:**
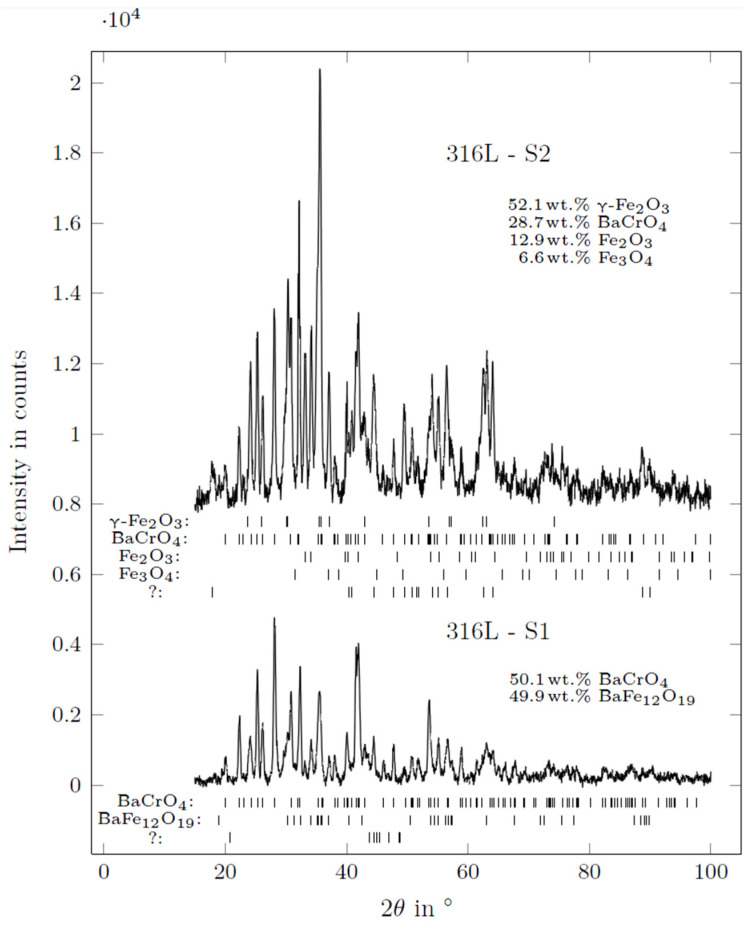
Diffraction patterns of the 316L (WE) after the electrochemical experiment at 850 °C, with liquid aluminum (RE) and the BaCO_3_ solid electrolyte: S1—green-colored reaction products; S2—brown-colored reaction products.

Figure 11 presents SEM micrographs of the reaction surface of the 316L (WE) with the positions of the performed EDS measurements marked. The corresponding results are listed in Table 6. Figure 12 presents the diffraction patterns of both mentioned reaction products found on the surface of the 316L (WE) (S1—green-colored reaction products; S2—brown-colored reaction products).

The microscopic analysis of 316L revealed a strong reaction of steel elements with the electrolyte during the experiment. Green-colored reaction products on the 316L (WE) surface (Figure 10a) measured on scan II (Figure 11) belonged to a Ba-Cr-O system and were indicated as BaCrO_4_ by XRD [32] (ICSD 62560) (Figure 12, S1). Scan II revealed the reaction products from the Ba-Fe-O system indicated as BaFe_12_O_19_ [33] (ICSD 87403). The brown-colored area (scan III) on the surface belonging to the detached reaction layer with leaf-shaped nanoparticles originated from the Fe-O system. Such leaf-shaped Fe-O nanostructures are commonly found in contact with salts under favorable polarization conditions [34,35]. In this area, the γ-Fe_2_O_3_ [36] (ICSD 87121) and BaCrO_4_, followed by Fe_2_O_3_ [37] (ICSD 15840) and Fe_3_O_4_ spinels [38] (ICSD 65341), were detected by XRD. It is presumed that the diffusion of iron into BaCO_3_ causes the formation of iron enrichment in the electrolyte at the vicinity of the electrode surface and thus the formation of such leaf-shaped structures. The surface of the electrode under the detached leaf-shaped iron oxide surface (scan IV) consisted mainly of pure iron oxide, with negligible amounts of Cr, Ni and Si but no Ba. Scans V–VIII were performed on selected agglomerates and crystal phases found on the surface of the electrode. Area V featured a partially oxidized Fe-Cr-Ni-Si agglomerate consisting mainly of iron. Scan VI identified the aluminum oxide residual from the refractory shielding pipe. The bright crystal phase (scan VII) was based on a Cr-Mn-Fe mixed oxide. The crystal phase of scan VIII was identified as Ba-Fe-Cr mixed oxide (most probably BaO·Cr_2_O_3_ and BaO·Fe_2_O_3_ [39,40]) with Mn within its structure. Goto and Takada [40] reported the existence of three compounds: BaO·5Fe_2_O_3_, BaO·2Fe_2_O_3_ and BaO·6Fe_2_O_3_ below 1000 °C when Fe_2_O_3_ was in contact with BaCO_3_. Figure 11d reveals numerous of those crystal phases on the green-colored reaction surface of the electrode (cf. Figure 10a).

**Figure 13:**
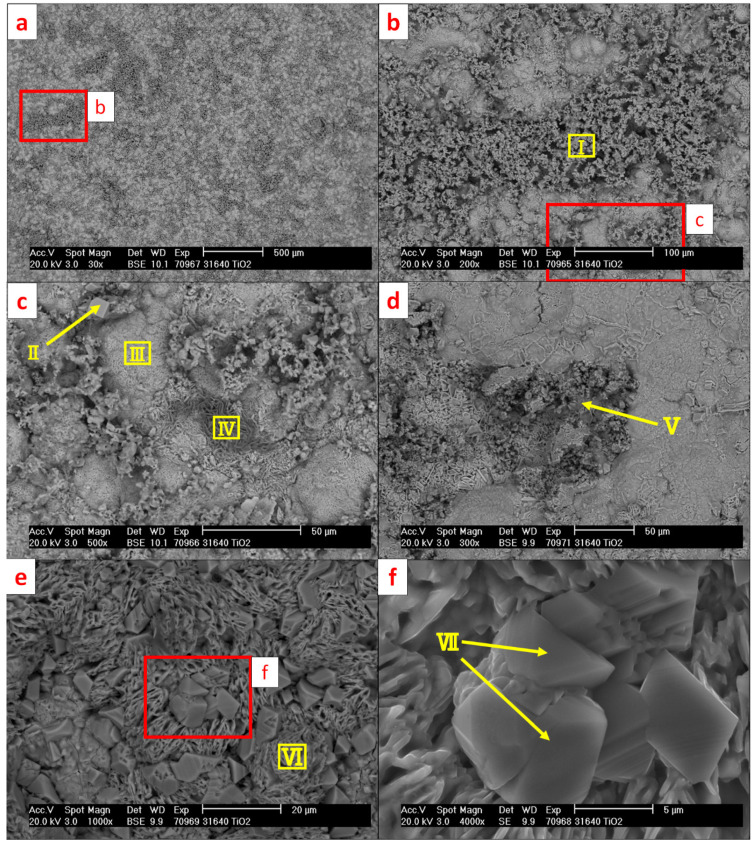
SEM micrographs (BSE mode) of 316L40TiO_2_ (WE) after the electrochemical experiment at 850 °C in the BaCO_3_ electrolyte with molten AlSi7Mg0.3 (RE): (**a**) overview; (**b**) green-colored BaCrO_4_ agglomerate; (**c**) BaFe_12_O_19_ with Ba-Cr-O; (**d**) Fe-Ti-O mixed oxides; (**e**) Fe-Mn-O-based surface with Fe-Mn-Ti-O based crystals; (**f**) high magnitude view of Fe-Mn-Ti-O crystals. Red frames indicate the regions of the magnified views of the micrographs; yellow frames and arrows indicate the areas of the EDS scans.

**Figure 14:**
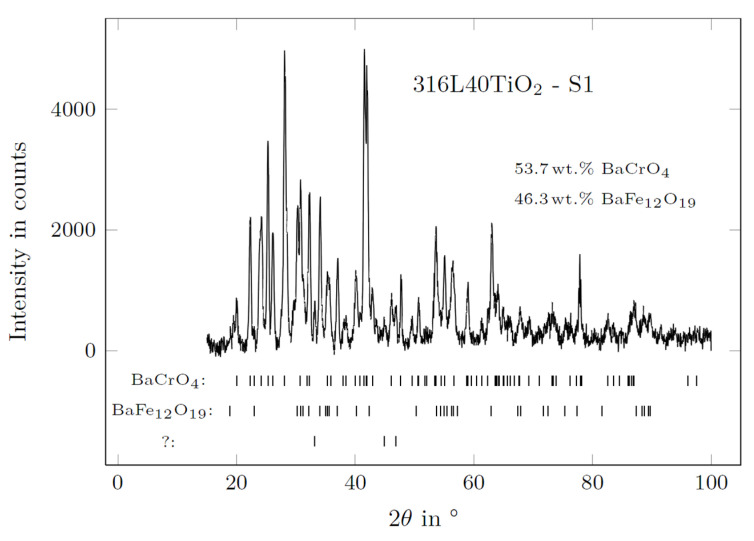
Diffraction pattern of the S1 reaction product surface of 316L40TiO_2_ (WE) after the electrochemical experiment.

Figure 13 presents SEM micrographs of the reaction surface of the 316L40TiO_2_ (WE) with the positions of the performed EDS measurements marked. The corresponding EDS results are listed in Table 7. The diffraction pattern of the well-distributed green-colored reaction product S1 of the 316L40TiO_2_ (WE) is presented in Figure 14.

Scans I and III of 316L40TiO_2_ (WE) (Table 7) were similar to scans II and I of the 316L (WE) (cf. Table 6). The reaction products of 316L40TiO_2_ (WE) analyzed for scan I were indicated by XRD as BaCrO_4_. In scan III, the products, consisting mainly of Ba, Fe and O, was indicated as BaFe_12_O_19_. Scan IV revealed a phase based on an Fe-Na-Mn-O system with negligible Ba content. Scan V exhibited the agglomerates based on Fe-Ti-O mixed oxides, presumably Fe-Ti-O spinels [39,41,42]. Such Fe-Ti-O spinels, e.g., titanomagnetite, have been found in natural environments; they start to form from 800 °C [43]. Scan VI exhibited an iron oxide nanoparticle structure with slightly different morphology in comparison to the iron oxide nanoparticle structure observed on the surface of 316L (WE) (cf. Figure 11b). Scans II and VII were performed on crystals found at the reaction surface of the 316L40TiO_2_ (WE). The composition of these crystals was similar to that of the surface on which they were formed. Scan II presented the crystal phase based on the Ba-Cr-O system found on the green-colored reaction surface (scan I). Scan VII contained no barium and was based on an Fe-Mn-Ti-O system. The clusters of such crystal phases are presented in Figure 13e, revealing the Fe-Mn-O-based surface indicated by scan VI.

The SEM/EDS and XRD analyses revealed BaCrO_4_ and BaFe_12_O_19_ in the green-colored-reaction-product areas of both working electrodes. The main elements that diffused into BaCO_3_ were Fe, Cr and Mn. Only a negligible amount of Ni was found in the analyzed reaction products in the SEM analysis of the surface of 316L40TiO_2_ (WE). The analysis of the brown-colored S2 surface of 316L (WE) revealed, besides the already mentioned BaCrO_4_, iron oxides with three different structures: α-FeO, γ-FeO and Fe_3_O_4_ spinel structures [44]. On the surface of 316L40TiO_2_ (WE), multiple Ti-containing crystals were found, confirming the participation of TiO_2_ in the formation of reaction products with the BaCO_3_ electrolyte and thus an impact on the electrochemical behavior of the composite electrode. All XRD patterns contained minor peaks which could not be matched to distinguished phases and, due to a lack of further information, remained undetermined.

### 3.5. XRD of BaCO_3_ in Contact with the AlSi7Mg0.3 Reference Electrode

Taking into consideration that the oxidation of the iron as well as the formation of BaCrO_4_ and BaFe_12_O_19_ occurred with the contribution of oxygen from the electrolyte, a decay of BaCO_3_ should have been observed. This commonly proceeds through the following reaction: BaCO_3_→BaO + CO_2_↑, or, taking into consideration that oxygen and barium are incorporated into reaction products, through: BaCO_3_→Ba^2+^ + O_2_↑ + CO↑. However, no residuals of Ba or BaO were found by the XRD analyses of the surfaces of the working electrodes.

**Figure 15:**
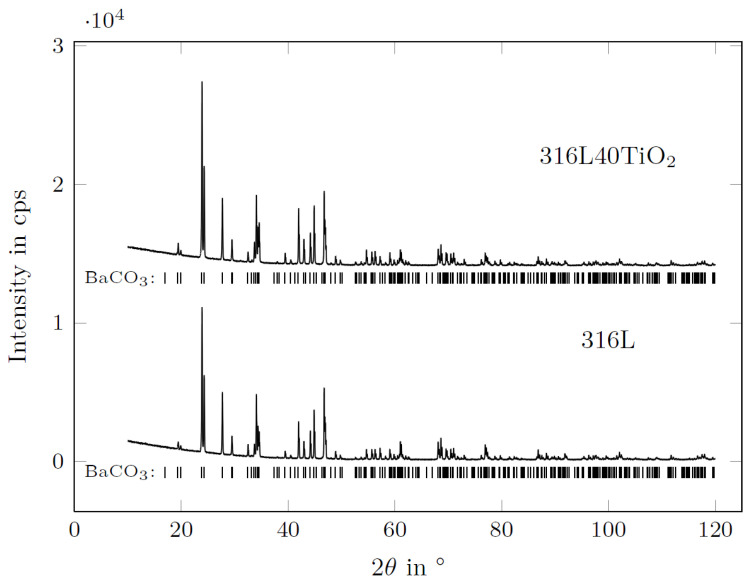
Diffraction pattern of BaCO_3_ after the electrochemical experiments with the 316L- and the 316L40TiO_2_-based systems.

The diffraction pattern of the BaCO_3_ electrolyte at the interface of the electrolyte and AlSi7Mg0.3 (RE) is presented in Figure 15.

The XRD measurement of BaCO_3_ after the experiment did not reveal any distinguishable decomposition or reaction of the electrolyte in contact with the liquid AlSi7Mg0.3 (RE). According to the Rietveld analysis of the BaCO_3_ powder (electrolyte), no other phases could be found, and only the diffraction pattern of BaCO_3_ was detected [45]. The missing BaO pattern indicates that no measurable decomposition of the BaCO_3_ occurred at elevated temperatures under polarization conditions in contact with the aluminum alloy (RE). Moreover, no infiltration of BaCO_3_ by the liquid aluminum alloy took place.

## 4. Conclusions and Outlook

A novel high-temperature electrochemical method was successfully developed and applied to determine the potential difference between a 316L stainless steel and a 316L stainless steel-TiO_2_ composite against a liquid AlSi7Mg0.3 aluminum alloy in a solid-state BaCO_3_ electrolyte under extremely demanding thermal conditions. Electrochemical cells were stabilized for more than 30 h at the designated test temperature of 850 °C. The potential differences between the working and reference electrodes were measured throughout the whole experiment, and their values corresponded to the open circuit potentials (*E*_Corr_s) of both electrodes determined by potentiodynamic polarization. The open circuit potentials of the 316L and 316L40TiO_2_ composite electrodes were 0.93 V and 0.71 V, respectively. The potentiodynamic polarization of the electrodes revealed no passivation behavior. Impedance spectroscopy, prior and posterior to the polarization, was successfully performed and revealed that solid BaCO_3_ exhibits suitable electrical properties as an electrolyte for high-temperature applications. The polarization resistance of the BaCO_3_ electrolyte was 100 Ω. The resistance of the electrode interface determined by impedance spectroscopy was determined as 53.6 kΩ for the 316L electrode and 81.9 kΩ for the 316L40TiO_2_ electrode. The effective capacitances were 21.56 pF and 8.73 pF for the 316L and 316L40TiO_2_ electrodes, respectively. The impedance characteristics were clearly more dispersed after the potentiodynamic polarization, but all parameters of the curve fitting remained unchanged.

The microscopic analysis of the working electrodes revealed vast diffusion of the electrode material into the BaCO_3_ electrolyte. The performed SEM/EDS and LSM analyses exhibited mixed oxides formed at the surfaces of the working electrodes, consisting mainly of barium and iron and, to lesser extents, chromium and manganese. In the case of the 316L40TiO_2_ sample, Ti was also found at the reaction surface, indicating the participation of TiO_2_ ceramic particles in the formation of the reaction phases and thus its influence on the differential potential between the composite and molten AlSi7Mg0.3. The XRD analyses of both samples’ surfaces revealed the existence of BaCrO_4_ and BaFe_12_O_19_ phases at the electrolyte–working-electrode interface. The XRD analysis of BaCO_3_ revealed no decomposition of the electrolyte in contact with the molten aluminum alloy. Hence, the suitability of BaCO_3_ for electrochemical trials with liquid-aluminum-alloy electrodes was proven. The elaborated electrochemical method can, after minor adaptation, be successfully applied for the determination of the differential potential and thus, also, the corrosion driving forces of any dissimilar liquid–solid or liquid–liquid metallic material pairs, under extremely demanding thermal conditions.

The study presents a method of measurement for the differential electrode potential arising between 316L stainless steel or a 316L + 40 vol% TiO_2_ composite and liquid AlSi7Mg0.3 at 850 °C, which can be directly related to the ion transfer between these materials. Nevertheless, to investigate the influence of the differential electrode potential on the ion transfer current, further studies of the system with the application of a molten AlSi7Mg0.3 aluminum alloy as the counter electrode will be indispensable.

## Figures and Tables

**Table 1:** Composition of the 316L stainless-steel powder (in wt.%) [1].

Steel	Fe	Cr	Ni	Si	Mo	Mn	Ti	Nb	S	Al
316L	Bal.	17.6	10.9	0.5	2.66	0.2	0.01	0.01	0.01	0.04

**Table 2:** Particle sizes and densities of the raw materials.

Raw Material	Particle Size in µm			True Density in g·cm^−3^
	D_10_	D_50_	D_90_	
316L	4	30	53	7.94
TiO_2_	0.1	1.9	4	4.30

**Table 3:** Composition of the AlSi7Mg0.3 aluminum alloy (in wt.%) [1].

Alloy	Al	Si	Mg	Fe	Cu	Mn	Zn	Ti	Cr	Ni
AlSi7Mg0.3	92.30	7.17	0.27	0.081	0.002	0.002	0.007	0.12	0.001	0.003

**Table 4:** Impedance results for the 316L (WE) and 316L40TiO_2_ (WE) systems in BaCO_3_ (electrolyte) at 850 °C in reference to the molten aluminum alloy AlSi7Mg0.3 (RE) prior to the potentiodynamic polarization.

Working Electrode	*R*_e_ in Ω	*R*_i_ in kΩ	*Q*_i_ in S*s^α^	*α*_i_	*C*_eff_ in pF	*τ*_eff_ in ns
316L	100	53.6	3.50 × 10^−10^	0.86	21.56	2.15
316L40TiO_2_	100	81.9	5.60 × 10^−10^	0.8	8.73	0.87

**Table 5:** Impedance curve-fitting results for the 316L (WE) and 316L40TiO_2_ (WE) systems in BaCO_3_ (electrolyte) at 850 °C in reference to the molten aluminum alloy AlSi7Mg0.3 (RE) posterior to the potentiodynamic polarization.

Working Electrode	*R*_e_ in Ω	*R*_i_ in kΩ	*Q*_i_ in S*s^α^	*α*_i_	*C*_eff_ in pF	*τ*_eff_ in ns
316L	100	53.6	3.50 × 10^−10^	0.86	21.56	2.15
316L40TiO_2_	100	81.9	5.60 × 10^−10^	0.8	8.73	0.87

**Table 6:** Results of the EDS scans of 316L (WE) indicated in Figure 11.

No.	Composition in at. %									
	O	Ba	Fe	Cr	Ni	Mo	Mn	Al	Si	Na
Ⅰ	57.9	15.1	23.6	-	-	-	0.7	-	0.9	1.8
Ⅱ	62.4	18.3	2.7	13.1	-	0.4	-	3.1	-	-
Ⅲ	41.6	2.3	50.8	-	0.5	-	2.0	-	1.3	1.5
Ⅳ	45.1	-	52.2	1.1	0.6	-	0.3	-	0.7	-
Ⅴ	14.5	-	69.2	5.7	5.3	0.7	0.2	-	4.4	-
Ⅵ	53.3	-	0.7	0.7	-	-	0.2	43.4	1.1	0.7
Ⅶ	66.4	-	6.1	16.8	0.4	-	10.3	-	-	-
Ⅷ	74.0	15.5	4.8	4.4	-	-	1.3	-	-	-

**Table 7:** Results of the EDS scans of 316L40TiO_2_ (WE) indicated in Figure 13.

Scan Area	Composition in at. %										
	O	Ba	Fe	Cr	Ni	Mo	Mn	Ti	Al	Si	Na
Ⅰ	63.2	19.7	1.5	10.1	-	1.7	0.5	-	0.5	0.1	2.7
Ⅱ	70.6	12.9	0.5	11.4	-	1.8	0.1	1.8	-	-	0.9
Ⅲ	51.1	16.3	26.9	-	-	0.4	0.2	2.1	-	1.2	1.8
Ⅳ	48.1	1.4	28.5	-	1.5	-	7.6	0.3	-	0.3	12.3
Ⅴ	45.9	-	30.8	0.2	-	-	3.4	15.7	-	-	4.0
Ⅵ	50.1	-	47.4	-	-	-	2.5	-	-	-	-
Ⅶ	58.3	-	24.7	-	-	-	8.2	4.6	-	-	4.2

## Data Availability

The data that support the findings of this study are available from the corresponding author upon reasonable request.
